# A Rare Case of Concrescence of Mandibular Third Molar and Supernumerary Fourth Molar

**DOI:** 10.1155/2022/3771299

**Published:** 2022-08-31

**Authors:** Jiao Wang, Errui Wang, Xin Yang, Lu Yuan, Zhige Li, Jie Zhang, Baoping Zhang

**Affiliations:** ^1^School of Stomatology, Lanzhou University, Lanzhou 730000, China; ^2^Department of Oral and Maxillofacial Surgery, Lanzhou University, Lanzhou 730000, China

## Abstract

Concrescence reveals a rare developmental anomaly in which two fully formed teeth are joined along the root surfaces by cementum, and generally occurs in maxillary molars, especially in a third molar and a supernumerary tooth. Very few cases have been reported about the concrescence of a third molar and a supernumerary fourth molar. Based on our available knowledge, this case report described a rare presentation in which concrescence is observed between a third molar and a supernumerary fourth molar in the mandible by diagnosing with cone-beam CT and histological examination.

## 1. Introduction

Aberrant gene regulation from ectodermal and mesenchymal tissues leads to developmental tooth abnormalities. According to morphological diversification, they are generally classified gemination, fusion, concrescence, syndontia and double teeth [1, 2]. For depth profiling phenotypes for these abnormalities, a previous study has found that tooth development depends on the interaction between the dental epithelium and the underlying ectomesenchyme [3].

Concrescence is a twinning anomaly involving the union of two teeth by cementum only. Such cases were frequently noted in maxillary molars, but were rarely found in the mandible, especially in a third molar and a supernumerary fourth tooth [2]. Previous research studies reported that the incidence of supernumerary fourth molar is 0.32%, not significantly different between the sexes [4], and the concrescence of teeth shared similar anatomic morphology with normal molars; usually it might not be erupted [5]. For clinical applications, it is important to establish a normative diagnosis so that proper treatment can be provided at the earliest time possible. This case reported a rare presentation in which concrescence was observed in a third molar and a supernumerary fourth molar of the mandible, and through appropriate treatment, it will provide a kind of standardized diagnosis code and acceptable therapeutic strategy.

## 2. Case Report

**Figure 1:**
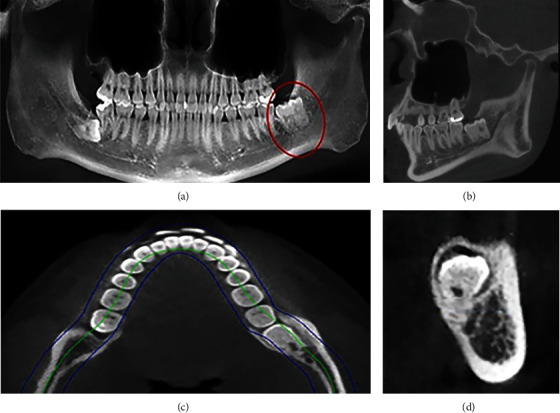
Preoperative CBCT examination. (a) A fourth molar in the mandible left third molar distal; (b–d) the relationship between roots and mandibular canal in coronal, transversal, and sagittal planes.

One year ago, a 21-year-old man extracted his right upper wisdom tooth in another hospital and found his third molar combination with fourth molar in the left mandibular after diagnosing with cone-beam computed tomography (CBCT). One day, he came to the Department of Oral and Maxillofacial Surgery of Hospital of Stomatology, Lanzhou University, with the chief complaint of hurt when chewing with the lower left posterior tooth. His medical history revealed no important health problems or traumas. His family members declared that they did not have any dental anomalies. Gingivitis and class I molar relationship was detected in intraoral examination. No wisdom teeth eruption, bleeding, or purulent secretion in mouth was observed. CBCT radiograph revealed that teeth 38# and 39# were vertical median impaction in the left mandibular region, and showed a concrescence between an impacted third molar and a supernumerary fourth molar (Figure 1) they shared similar shape but self-governed root canal system. Besides, teeth 48# was impacted at horizontal-low level (Figure 1).

### 2.1. Preoperative Preparation

Before surgery, we conducted a comprehensive assessment of the risk of surgery based on the patient's general condition and CBCT examination. The evaluation criteria were based on the Chinese Stomatological Association's assessment of the risk factors for lower alveolar nerve injury related to the extraction of mandibular third molars [6] (Table 1). The patient's temporary inferior alveolar nerve injury (IANI) score was divided into 4 levels, permanent IANI was scored as 4 points, and the surgical risk of this case was high hazard classification. The risks of IANI include low hazard classification (≤2 points), moderate hazard classification (3 points) and high hazard classification according to the score (≥4 points and ≤6 points) [6]. Furthermore, according to the preoperative discussion on flap design and incision design, we finally chose the envelope flap, which was proposed by Gustav Otto Kruger [7]. During surgery, a gingival sulcus incision was made from the mesial side of the mandibular second molar to the mesial surface of the mandibular second molar near the buccal tip, and then the incision was extended 45° obliquely to the mandibular ramus, which was helpful to reduce postoperative swelling and pain [8] and in turn beneficial to postoperative recovery.

### 2.2. Treatment Process

**Figure 2:**
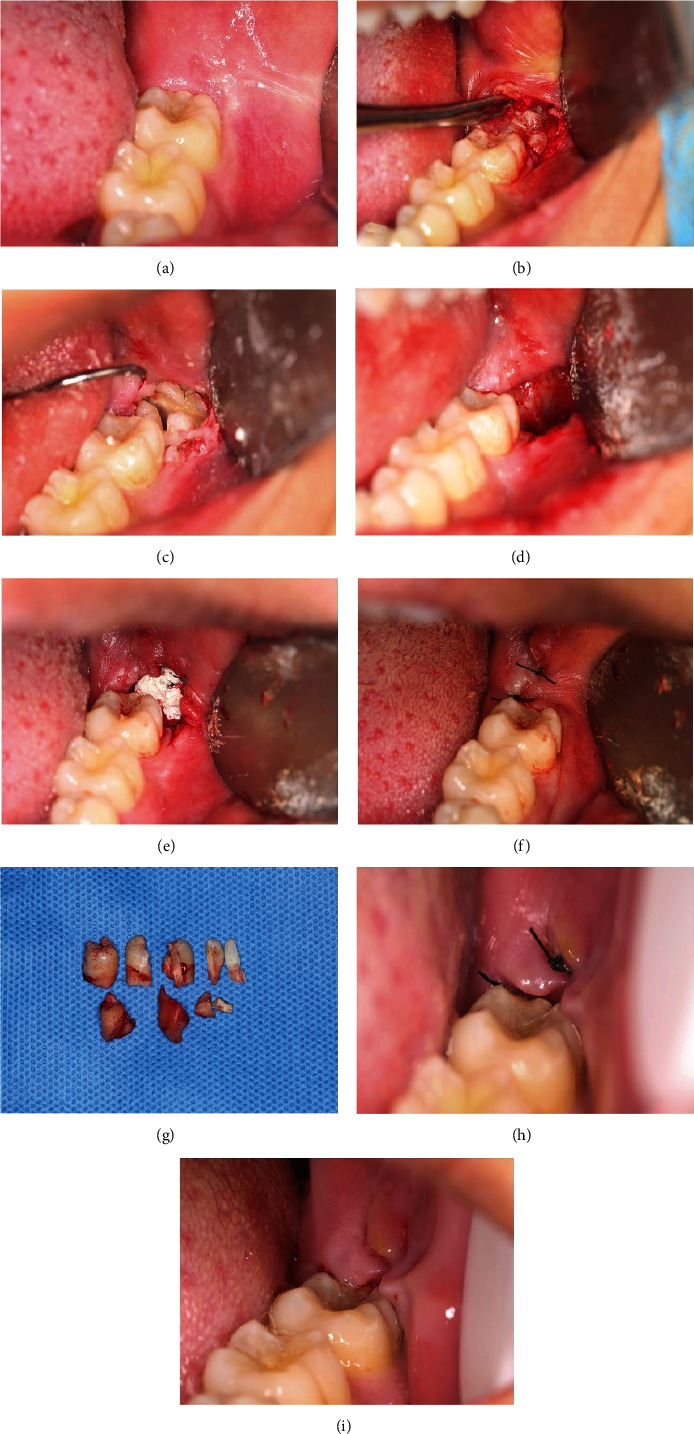
Surgery process and follow-up. (a) No wisdom teeth erupting, bleeding, or purulent secretion in the mouth. (b–f) Teeth extraction and alveolar reconstruction. (g) The fragment of teeth. (h, i) Removed stitches without severe complication and the defect of the alveolar socket healed well.

The patient enrolled for removal of the concrescence teeth. Surgery was carried out under local lidocaine hydrochloride (2%, 2107301, Tianjin Jinyao Pharmaceutical Co, China) and articaine (4%, S-124, Acteon group France, France)-based anesthesia blocking the inferior alveolar, lingual, and buccal nerve. A mucoperiosteal flap was prepared and raised, and then ostectomy and odontomy were performed using handpiece (Figure S1) and sterile saline irrigation. The third and fourth molars were removed. The entire procedure went very smoothly with minimal bleeding and no exposure of the inferior alveolar nerve. After curettage of the cavity, the socket was flushed with saline, and stuffed large alveolar fossa defects with artificial bone material (20201216101B, Beijing Aojing Pharmaceutical Technology Co, China). Finally, the flap was repositioned with 2-0 silk sutures (220103, Ningbo Medical Suture Co, China) (Table S1). Antiflogistic and analgesics therapy were applied for 3–5 days (Figures 2(a)–2(g)).

### 2.3. Postoperative Follow-Up

After 7 days, the sutures were removed without severe complications (Figures 2(h)–2(i)). The envelope flap incision design mentioned above significantly reduced postoperative swelling. In the intraoral photograph one week after the operation, we could see the soft tissue coverage of the 37# distal gingival without obvious swelling. Oral clinical examination: no numbness, paresthesia, pain, and loss of touch were noted in the left lower lip and jaw. There is also no numbness in the ipsilateral half tongue.

**Figure 3:**
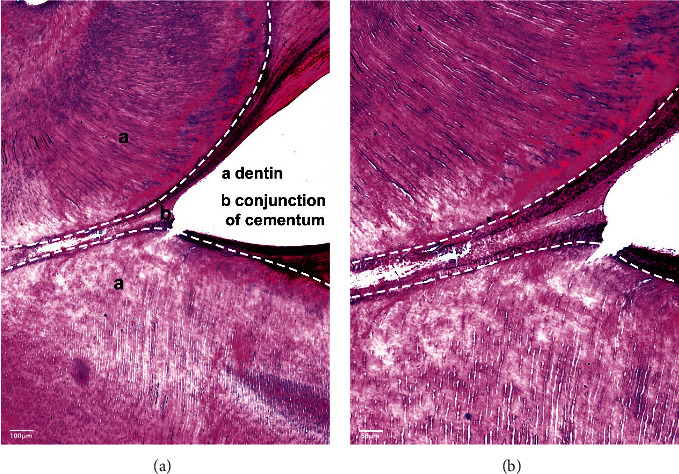
Photomicrographs of histological sections stained with the hematoxylin–eosine stain exhibiting the point of cementum fusion, revealing a real concrescence. Images obtained using (a) a 10x microscope and (b) a 20x microscope.

Histological sections were employed to evaluate which odontogenic tissues were involved in the affected teeth. The histological examination revealed fusion of cementum between the mandibular third molar and supernumerary fourth molar which is diagnostic for concrescence (Figure 3).

**Figure 4:**
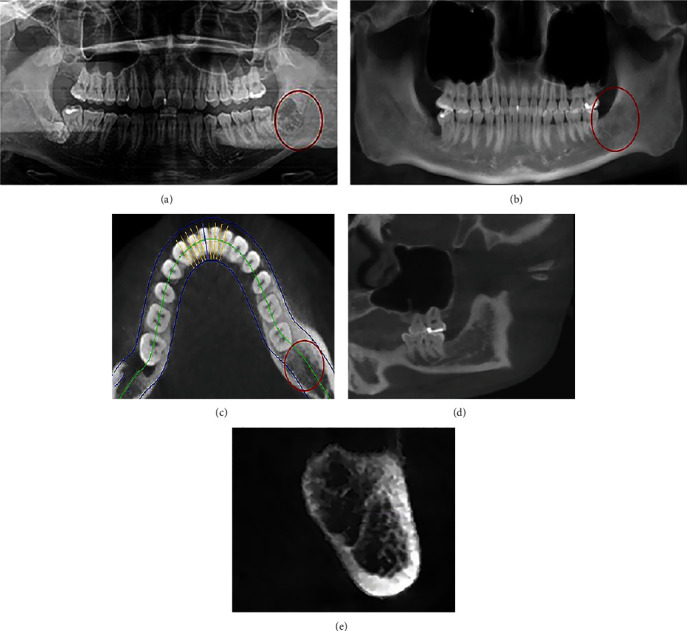
(a) Immediate postoperative panoramic examination showed the empty tooth socket; (b) after six months, CBCT examination showed good bone formation, and the alveolar bone healed well. (c) Transverse section shows increased bone density in empty alveolar bone; (d, e) Coronal and sagittal plane shows increased height of the alveolar bone.

Oral clinical examination after 6 months: there was no swelling in the distal mucosa of the 37# and the 37# distal periodontal attachment was in good condition. CBCT examination showed increased bone density (Figure 4).

## 3. Discussion

Concrescence may occur during root formation or after the radicular phase of development is completed. From the stages of tooth development, different degrees union of cementum, dentine, and enamel are possible [2], and the cause of tooth development abnormalities is hyperactivity of odontogenic epithelium [9]; the failure of the process of degeneration of the residual dental lamina cells leads to further reaction and over-proliferating and eventually develops into supernumerary teeth [10].

Teeth fusion is a condition in which two separate tooth buds have a joined crown that resembles a bifid crown, but concrescence is a form of fusion in which the union is in the cementum alone without confluence of the underlying dentine. In practice, there are very few cases about the concrescence of a third molar and a supernumerary tooth [11]. CBCT radiograph in the case showed that the roots of 38# and 39# seem to be connected, but their dentin was separated and their root canal system was independent. Histological examination revealed fusion of cementum between the mandibular third molar and supernumerary forth molar. In summary, concrescence of teeth (38# and 39#) was diagnosed. Slight bone defects were found in the distal of 37#. According to these findings, a surgical treatment option was provided to deal with concrescence of teeth.

Based on a plenty of the literature studies, it was found that the maxillary fourth molar is more common than those in the mandible, most of which cannot erupt, have none symptoms, and can be found by radiology examinations accidently [12]. Local infection factors, trauma, space restriction in the development, or excessive occlusal force plays crucial roles in phenotypic anomalies. It is very difficult to detect concrescence clinically, and radiographic examination is a means to distinguish between concrescence and other pathological state. When the clinical examination points to affecting aesthetics, causing crowded dentition, eruption pathologies or tooth displacement, periodontal disease, pericoronitis, root resorption of adjacent teeth, bone resorption, or cyst formation [13], treatment is required, or subjective to wishes of the patient, the teeth are supposed to be removed in time.

Several approaches are available for the treatment of supernumerary concrescence. The treatment can be performed in two means: (a) surgical extraction or (b) maintenance of the asymptomatic tooth and periodic monitoring at least once a year [9]. In this case, the impacted teeth led to pericoronitis sometimes, and they might cause the absorption and destruction of the distal neck bone in 37#. Also, the patient had the subjective willingness of extraction. Therefore, we extracted the 38# with 39# and reconstructed the alveolar with artificial bone material to preserve the bone in the distal second molar. The defected alveolar socket appeared well-healed in CBCT examination six months after treatment. In the literature, there have been rare case reports of successful surgical division of concrescent teeth, or selected shaping with or without placement of full crowns has been used for the special cases. This case report is rarely found in the mandible in which concrescence has been observed between a third molar and a supernumerary fourth molar with the space-occupying bone filling materials, restoring the massive bone defect after extraction, and it will provide a reference for the management of similar cases of supernumerary fourth molars.

## Figures and Tables

**Table 1:** Construction of a scoring system for predicting preoperative risk of inferior alveolar nerve injury (IANI).

Variable	Classification	Score	
		Temporary IANI	Permanent IANI
Age	<25 years old	1	1
Gender	Female	0	0
Contact relationship between mandibular third molar and IAC	Contact	2	2
The vertical contact position of the mandibular third molar with IAC	Middle 1/2 of the root to the neck of the tooth	0	0
Impaction depth	Low-level hindrance	0	0
Root number	Single/multiple	1	1
Total		4	4

Note: IAC, inferior alveolar canal. Risk factors for IANI include the number of roots of mandibular third molars (*P* < 0.01), the impacted depth (*P* < 0.05), and the contact relationship between the roots and the IAC (*P* < 0.01) and vertical contact location (*P* < 0.05). Age <25 years was an independent risk factor for temporary IANI (*P* < 0.001), being female is a risk factor for permanent IANI (*P* < 0.05).

## Supplementary Materials

Supplementary Materials Figure S1: The hand piece and drills used for bone removal and for tooth sectioning. A: Drills for tooth cutting; B: Hand piece for tooth sectioning. Table S1: Medicines, materials and equipment used during surgery.
