# Supplementary material for: A Rare Case of Concrescence of Mandibular Third Molar and Supernumerary Fourth Molar
Source: Case Rep Dent. 2022 Aug 31;2022:3771299. doi: 10.1155/2022/3771299 (PMC9452987; doi:10.1155/2022/3771299)
Supplement: Supplementary Materials — Figure S1: The hand piece and drills used for bone removal and for tooth sectioning. A: Drills for tooth cutting; B: Hand piece for tooth sectioning. Table S1: Medicines, materials and equipment used during surgery. [file 3771299.f1.docx]

**Supplementary Descriptions**


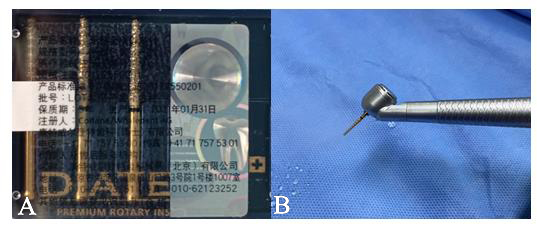


**Figure S1** The hand piece and drills used for bone removal and for tooth sectioning. A: Drills for tooth cutting; B: Hand piece for tooth sectioning.

**Table S1** Medicines, materials and equipment used during surgery.

|  | Production batch | Manufacture | Country |
| --- | --- | --- | --- |
| Lidocaine | 2107301 | Tianjin Jinyao Pharmaceutical Co. | China |
| Articaine | S-124 | Acteon group France | France |
| Drill | 2017255050201 | Conte Wildent Dental Trading Co. | Switzerland |
| Hand piece | X17120300 | Sino Medical Equipment Group Co. | China |
| Artificial bone material | 20201216101B | Beijing Aojing Pharmaceutical Technology Co. | China |
| Suture | 220103 | Ningbo Medical Suture Co. | China |
